# Variation in the Use of Orbital Atherectomy During Coronary Artery Intervention in the United States

**DOI:** 10.1016/j.jscai.2022.100529

**Published:** 2022-11-26

**Authors:** Anna K. Krawisz, Abby M. Pribish, Kevin Kennedy, Eric A. Secemsky

**Affiliations:** a Richard A. and Susan F. Smith Center for Outcomes Research in Cardiology, Department of Medicine, Beth Israel Deaconess Medical Center, Boston, Massachusetts;; b Harvard Medical School, Boston, Massachusetts;; c Division of Cardiology, Department of Medicine, Beth Israel Deaconess Medical Center, Boston, Massachusetts;; d Division of Cardiology, University of Colorado School of Medicine, Aurora, Colorado;; e Mid America Heart Institute, Kansas City, Missouri

**Keywords:** atherectomy, complex percutaneous coronary intervention, coronary artery calcification

More than 30% of patients who undergo percutaneous coronary intervention (PCI) have moderate to severe coronary artery calcification (CAC), which is associated with procedural failure and adverse outcomes.^1^ Moreover, the prevalence of CAC is likely to increase as the major risk factors for CAC—diabetes, advanced age, and chronic kidney disease—are increasing.^2^ Therefore, there is a critical need to improve outcomes in patients with CAC.

Two predominant atherectomy techniques exist to modify CAC before coronary stent implantation. Rotational atherectomy (RA) has been used for the previous 3 decades. Despite being the most frequently used atherectomy technique, it has not been shown to confer a clinical benefit. More recently, orbital atherectomy (OA) has been introduced. OA has key differences from RA in its mechanism of action; however, there is a paucity of data regarding its use and associated clinical outcomes. There is no standardized approach to the treatment of CAC with atherectomy, and there are no trials comparing RA and OA head-to-head. Hospitals and interventionalists determine whether to use atherectomy and which device to use. Therefore, there is likely to be significant variation in the use of atherectomy among hospitals. In hospitals that have both available, the decision to select RA versus OA has not been well characterized.

Given the evolution of interventional therapies for coronary artery disease and the significant impact that CAC has on PCI outcomes, it is critical to better understand how contemporary atherectomy devices are being applied at a national level. In this analysis, we leverage the Healthcare Cost and Utilization Project (HCUP) National Inpatient Sample database to answer the following 2 questions. First, which patient populations are being treated with OA versus RA in contemporary practice? Second, are there differences in the use of OA versus RA among hospitals at which both devices are used?

Patients from the HCUP National Inpatient Sample who underwent PCI in 2016 or 2017 were included. Patients who underwent PCI and atherectomy were identified using *International Classification of Diseases, 10th Revision*, Procedure Coding System codes. The following codes identified atherectomy: 02C03ZZ, 02C13ZZ, 02C23ZZ, 02C33ZZ, 02C03ZZ, 02C13ZZ, 02C23ZZ, and 02C33ZZ for RA and X2C0361, X2C1361, X2C2361, and X2C3361 for OA. Baseline characteristics were ascertained using Elixhauser comorbidities. The HCUP National Read-missions Database was used to identify patients who required readmission.

All metrics and normally distributed variables are reported as means ± standard deviations and were compared using the *t* test. Categorical variables were compared using χ^2^ tests. A *P* value of <.01 was considered significant. The median odds ratio of the likelihood of undergoing atherectomy was calculated for the hospitals at which at least 10 atherectomy procedures were performed per year. SAS statistical software version 9.4 (SAS Institute) was used. The Institutional Review Board at Beth Israel Deaconess Medical Center evaluated this study and waived the need for approval because human subject research was not involved.

Of 135,645 patients who underwent PCI in 2016 and 2017, 15,153 (11.2%) underwent atherectomy. Of those, 1332 (8.8%) underwent OA and 13,821 (91.2%) underwent RA. Patients who underwent OA were significantly older (72.5 vs 64.6 years; *P* < .01) and more likely to be women (38.1% vs 28.4%; *P* < .01) than those who underwent RA. In addition, those who underwent OA versus RA had a significantly higher burden of comorbidities, including congestive heart failure (3.2% vs 1.7%; *P* < .01), chronic pulmonary disease (26.1% vs 17.3%; *P* < .01), peripheral vascular disease (18.1% vs 11.7%; *P* < .01), diabetes (51.0% vs 37.1%; *P* < .01), and renal failure (31.7% vs 17.8%; *P* < .01).

Interestingly, patients who underwent OA were significantly less likely to undergo PCI for an acute myocardial infarction (46.0% vs 81.2%; *P* < .01) and were significantly more likely to be admitted electively (24.5% vs 12.0%; *P* < .01) than those who underwent RA. Patients who underwent OA versus RA had longer lengths of hospital stay (5.6 ± 6.0 vs 4.8 ± 6.9 days; *P* < .01) and were more likely to be readmitted (16.1% vs 12.4%; *P* < .001). Those who underwent OA versus RA were disproportionately more likely to be in metropolitan areas with a population of one million people or greater.

During the study period, a yearly average of 34 PCIs involving atherectomy were performed at hospitals. In a cohort of hospitals at which 10 or more PCIs with atherectomy were performed per year, no OA procedures were performed at 49% of hospitals (Figure 1). The median odds ratio for undergoing OA at a hospital that facilitated both RA and OA was 2.76 (interquartile range, 2.54–2.97; *P* < .01), demonstrating a large amount of interhospital variability in the use of OA. Of the sites that perform both OA and RA, OA procedures were the minority of atherectomies performed, with few sites using OA in 50% or more of all PCIs requiring atherectomy.

There were significant differences in the geographic distribution of OA. Although the proportions of OA and RA performed in the South were similar (37.0% vs 38.3%), proportionally more OAs were performed in the Northeast (29.0% vs 19.1%) and fewer in the Midwest (18.0% vs 23.1%) and West (16% vs 19.5%). Proportionally more OAs were performed in highly populated, metropolitan areas compared with RAs (36.8% vs 28.7%). In addition, a higher proportion of OAs were performed in urban teaching hospitals (82.1% vs 77.6%). There was no statistically significant difference in the proportions of OA and RA performed in hospitals with different bed sizes.

In this nationwide analysis, we found substantial differences in the use of OA compared with that of RA. First, OA is being used significantly less than RA and comprised only 9% of coronary atherectomies in our study. Second, OA is being used in a significantly different population than the population in which RA is used. We found that patients who underwent OA are more chronically ill with a much higher burden of comorbidities but are less acutely ill with significantly lower levels of acute myocardial infarction at the time of atherectomy than those who underwent RA. Of note, the analysis demonstrated the same pattern of results when OA from high OA-facilitating sites were compared with RA from high RA-facilitating sites, sites which performed the top 15% of OA and RA procedures, respectively. Third, there is significant interhospital variation in the use of OA. Simply being treated at a different hospital dramatically changed the odds of undergoing OA, irrespective of case indication or patient comorbidities. The use of OA also varied by geography and location (rural or urban).

These results highlight the lack of a standardized approach to coronary atherectomy use. This is likely the result of the paucity of randomized trials that have evaluated outcomes related to atherectomy use and the lack of head-to-head comparison between atherectomy devices. Although small trials have shown promise for OA,^3^ we await randomized-controlled trial data from completion of the Evaluation of Treatment Strategies for Severe Calcific Coronary Arteries: Orbital Atherectomy vs Conventional Angioplasty Technique Prior to Implantation of Drug-Eluting Stent trial, which is powered to assess the superiority of OA vessel preparation compared with conventional angioplasty.^4^ In addition, given that OA was not approved until 2013, some of the variation observed in the analysis may be due to lower availability of OA technology or the time required to educate and train operators in using the technology.

There are several limitations to this study. First, our study population was primarily of White race, which limits the generalizability to other populations. Second, because the patient populations who underwent OA and RA were substantially different, we were unable to compare outcomes between OA and RA because of the high likelihood of confounding. Third, there were relatively low numbers of patients who underwent OA (1332 patients, 8.8% of total coronary atherectomy procedures). Fourth, claims data do not include the granular details regarding anatomic and lesion characteristics that may have impacted the choice of atherectomy device.

## Figures and Tables

**Figure 1.**
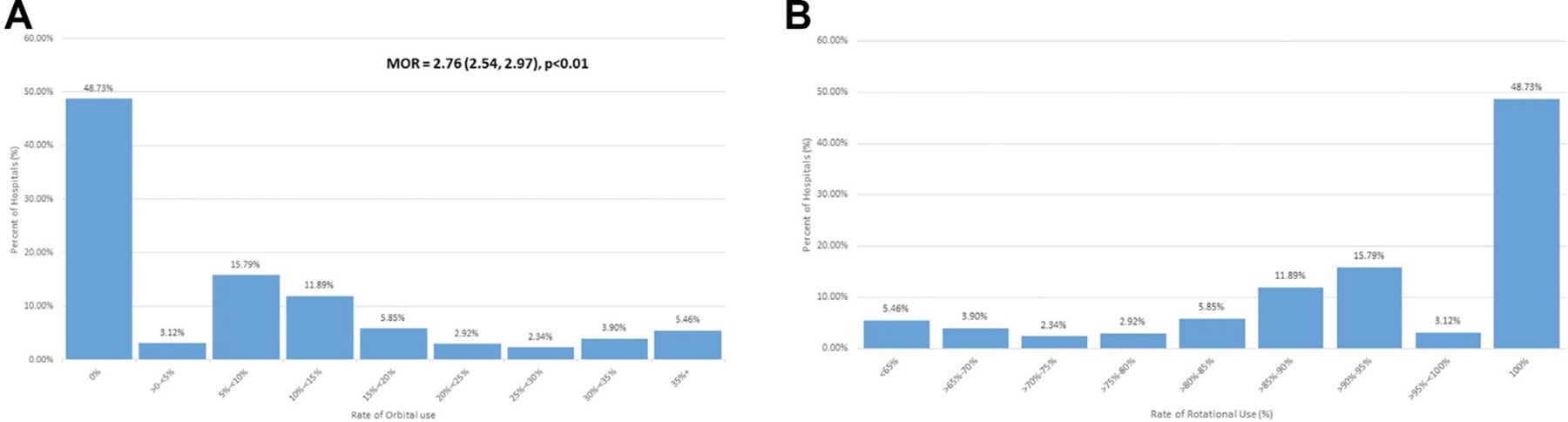
Interhospital variation in orbital atherectomy and rotational atherectomy use. The distribution of (**A**) orbital atherectomy (OA) and (**B**) rotational atherectomy (RA) use in hospitals that perform at least 10 coronary atherectomy procedures annually from 2016 to 2017 are displayed. (**A**) Almost half of the hospitals at which atherectomy is performed do not perform OA. The median odds ratio (MOR) for undergoing OA is 2.76 (interquartile range, 2.54–2.97; *P* <.01), demonstrating a large amount of interhospital variability in the use of atherectomy. (**B**) In contrast, the majority of hospitals facilitate RA, and in 49% of hospitals, RA comprises 100% of atherectomies performed.

## References

[1] Généreux P, Madhavan MV, Mintz GS, Ischemic outcomes after coronary intervention of calcified vessels in acute coronary syndromes. Pooled analysis from the HORIZONS-AMI (harmonizing outcomes with revascularization and stents in acute myocardial infarction) and ACUITY (acute catheterization and urgent intervention triage strategy) trials. J Am Coll Cardiol. 2014;63(18):1845–1854. 10.1016/j.jacc.2014.01.034 24561145

[2] Madhavan MV, Tarigopula M, Mintz GS, Maehara A, Stone GW, Généreux P. Coronary artery calcification: pathogenesis and prognostic implications. J Am Coll Cardiol. 2014;63(17):1703–1714. 10.1016/j.jacc.2014.01.017 24530667

[3] Chambers JW, Feldman RL, Himmelstein SI, Pivotal trial to evaluate the safety and efficacy of the orbital atherectomy system in treating de novo, severely calcified coronary lesions (ORBIT II). JACC Cardiovasc Interv. 2014;7(5):510–518. 10.1016/j.jcin.2014.01.158 24852804

[4] Evaluation of treatment strategies for severe calcific coronary arteries: orbital atherectomy vs. conventional angioplasty technique prior to implantation of drug-eluting stents: the ECLIPSE trial (ECLIPSE). ClinicalTrials.gov identifier: NCT03108456. Accessed February 1, 2022. https://clinicaltrials.gov/ct2/show/NCT03108456

